# S1PR2 deficiency enhances neuropathic pain induced by partial sciatic nerve ligation

**DOI:** 10.3906/sag-1710-77

**Published:** 2019-02-11

**Authors:** Qiang GU, Jin-Chao HOU, Xiang-Ming FANG

**Affiliations:** 1 School of Medicine, Zhejiang University, Hangzhou P.R. China; 2 Department of Anesthesiology, Affiliated Hospital of Nantong University, Nantong P.R. China; 3 Department of Anesthesiology, The First Affiliated Hospital, School of Medicine, Zhejiang University, Hangzhou P.R. China

**Keywords:** Neuropathic pain, S1PR2, partial sciatic nerve ligation, matrix metalloproteinase

## Abstract

**Background/aim:**

Sphingosine 1-phosphate receptor 2 (S1PR2), a member of the seven-transmembrane receptor family, can be activated by its natural ligand sphingosine 1-phosphate (S1P) to initiate signal transduction and is involved in a wide range of biological effects such as immune cell migration and vascular permeability. Its relationship with neuropathic pain (NP) has not been reported. In this study, the effects of S1PR2 on the development of NP were studied.

**Materials and methods:**

We generated a model of NP by partial sciatic nerve ligation (pSNL). The 50% paw withdrawal threshold of the wild-type (WT) group and the S1PR2 deficiency group were measured at several time points after surgery. The inflammatory factor levels of the two groups were measured by real-time quantitative polymerase chain reaction (RT-PCR). Neutrophil infiltration and glial cell activation were detected by immunofluorescence. Matrix metalloproteinase 9 (MMP9) and its substrate myelin basic protein (MBP) were measured by RT-PCR, western blotting, and immunofluorescence.**Result: **The S1PR2 deficiency group showed a reduction in 50% paw withdrawal threshold compared with WT mice (P < 0.05) at 3 days after the operation. In the ligated sciatic nerve of the S1PR2 deficiency group, the mRNA expression of IL-1β was increased; the numbers of infiltrating neutrophils and activated astrocytes were also increased. The expression of MMP9 was elevated while MBP was decreased.

**Conclusion:**

S1PR2 deficiency could increase the pain sensitivity of a NP mouse model and promote the development of NP.

## 1. Introduction

Neuropathic pain (NP) is defined as ‘‘pain caused by a lesion or disease of the somatosensory system’’, as stated by the International Association for the Study of Pain in 2011 (1). The point prevalence of chronic pain with neuropathic characteristics has been reported as 10.6% (95% CI: 9.75–11.45) (2). NP causes substantial disability, but current treatment is inadequate (3). Neuroimmune regulation in the pathogenesis of NP has attracted attention, and effective immune molecular targets for the treatment of NP are currently being sought.

Sphingosine 1-phosphate receptor 2 (S1PR2), a member of the seven-transmembrane (G-protein-coupled) receptor family, can be activated by its natural ligand, sphingosine 1-phosphate (S1P), to initiate signal transduction and is involved in a wide range of biological effects (4). It has been shown that S1PR2 can modulate immune cell migration (5) and vascular permeability (6) and mediate changes in neuronal plasticity (7). It has also been reported that immune cell infiltration (8), changes in vascular permeability, and synaptic plasticity of neurons can affect pain development (9,10). Therefore, the current study was undertaken to explore the effect of S1PR2 on NP. 

## 2. Materials and methods

### 2.1. Animals

All experiments were conducted in accordance with the ethical guidelines of the animal care and use committee of the relevant university (China). Adult C57BL/6 mice were purchased from Shanghai SLAC Laboratory Animal Co., Ltd. (Shanghai, China). S1PR2-KO mice were obtained from the Mutant Mouse Regional Resource Centers (USA; MMRRC Strain ID, 12830). The mice were maintained in a specific-pathogen-free mouse facility with food and water ad libitum and were kept at a constant ambient temperature of 24 ± 1 °C under a 12-h/12-h light/dark cycle. The S1PR2-KO mouse line was backcrossed with the C57BL/6 strain for at least seven generations, and the heterozygous offspring were intercrossed to produce wild-type (*S1pr2*+/+) and S1PR2-KO (*S1pr2*-/-) mice, which were used for the current investigation. Male mice aged 12–16 weeks were used in this study. All experiments were performed on 12- to 16-week-old mice. 

### 2.2. Pain models

All animals were housed in ventilated cages and were acclimated for at least 1 week before any experimental procedures. Both strains of mice were randomly divided between two groups: left-side pSNL and sham surgery only. 

A pSNL mouse model was generated by unilateral partial ligation of the sciatic nerve as described previously with slight modifications (11). Briefly, all animals were anesthetized with 1.5% to 2% isoflurane delivered via a modified nose cone. Then a 5-mm incision was made to expose the left sciatic nerve just distal to the branch leading to the posterior biceps femoris/semitendinosus muscles. The one-third to one-half diameter of the left sciatic nerve at the upper thigh level was ligated tightly with an 8-0 silk suture. Sham surgeries were also performed by exposing the sciatic nerve without ligation. The wound was closed by suturing the muscle and skin layers. The animals were allowed to recover in their cages before further testing.

### 2.3. Behavioral test

Animals were acclimatized to the testing room for at least 1 h before behavioral tests. The same experimenter handled and tested all animals in each experiment and was blinded to the genotype and surgical treatment of each animal.

Mechanical sensitivity was assessed by the up-down method using calibrated von Frey filaments as described previously with slight modifications (12). Mice were acclimatized on a metal mesh floor in small cylinders (diameter 7.5 cm; height 10 cm) for 2 h. Mechanical sensitivity was evaluated using a set of eight calibrated von Frey filaments (0.008, 0.02, 0.04, 0.07, 0.16, 0.4, 0.6, 1.0, and 1.4 g) that were applied to the plantar surface of the hind paw until the filament bent slightly for a few seconds. A withdrawal reflex of the hind paw during stimulation or immediately after stimulus removal was considered a positive response. The first stimulus was always the 0.16-g filament. When there was a positive response, the next lower filament was applied, and when there was no response, the next higher filament was used. After the first change in response, four additional responses were observed, and the 50% paw withdrawal threshold value was calculated. When a positive response to a stimulus with the 0.008-g filament or a negative response to a stimulus with the 1.4-g filament was observed, values of 0.008 and 1.4 g were assigned, respectively.

### 2.4. RT-PCR

The injured sciatic nerve of pSNL-induced NP model mice was cut 5 mm distal and proximal to the ligation site, and the section was removed. Lumbar spinal cord samples (L4–L6) were also harvested from the NP model mice. Total RNA was extracted from the harvested tissues (the injured sciatic nerve and the ipsilateral lumbar enlargement at L4–L6) using TRIzol Reagent (Invitrogen, Carlsbad, CA, USA). Complementary DNA was generated by reverse transcription of 1 µg of total RNA using the Reverse Transcription System (Promega, Madison, WI, USA). Quantitative measurements of the S1PR2, IL-1β, IL-6, TNF-α, MMP9, and MMP2 expression levels were performed using the ABI 7500 Fast Real-Time PCR system. A PCR volume of 20 µL contained 10 µL of SYBR Premix Ex Taq II, 0.4 µL of ROX Reference Dye II, 0.4 µL of each primer, and 0.4 µL of complementary DNA. Each sample was assayed in triplicate. The reaction conditions were as follows: heat activation for 10 min at 95 °C, followed by 40 cycles of 95 °C for 10 s, 60 °C for 30 s, and 72 °C for 30 s. The housekeeping gene β-actin was used as an internal control. Gene expression levels were determined using the 2–ΔΔCT relative quantification method. The primer sequences are shown in the Table. 

**Table 1:** The primer sequences used in RT-PCR.

Primer	Sequences
β-actin	Forward: 5’-GTGACGTTGACATCCGTAAAGA-3’ Reverse: 5’-GCCGGACTCATCGTACTCC-3’
S1PR2	Forward: 5’-CACTGCTCAATCCTGTCA-3’ Reverse: 5’-GAAATGTCGGTGATGTAGG-3’
IL-1β	Forward: 5’-GAAATGCCACCTTTTGACAGTG-3’ Reverse: 5’-TGGATGCTCTCATCAGGACAG-3’
IL-6	Forward: 5’-GCCTTCTTGGGACTGATG-3’ Reverse: 5’-AGGTCTGTTGGGAGTGGTA-3’
TNF-α	Forward: 5’-TACTGAACTTCGGGGTGA-3’ Reverse: 5’-ACTTGGTGGTTTGCTACG-3’
MMP9	Forward: 5’-GGCTGTGGTCTTATCTCCAAC-3’ Reverse: 5’-TGATGTTATGATGGTCCCACTTG-3’
MMP2	Forward: 5’-CAAGTTCCCCGGCGATGTC-3’ Reverse: 5’-TTCTGGTCAAGGTCACCTGTC-3’

### 2.5. Western blot

The injured sciatic nerves were collected and homogenized in RIPA lysis buffer containing a mixture of PMSF and other protease inhibitors (Beyotime, Shanghai, China). Protein samples were separated on a 12% Bis-Tris polyacrylamide gel (Novex, USA) and transferred onto polyvinylidene fluoride membranes (Millipore Corporation, USA). The membranes were blocked and then incubated overnight at 4 °C with a primary antibody [rabbit anti-β-tubulin, 1:1000 (Santa Cruz, Japan); rat antimyelin basic protein, 1:1000 (Abcam, USA); rabbit anti-MMP9, 1:1000 (Abcam, USA)]. The membranes were then incubated with the corresponding HRP-conjugated secondary antibodies for 1 h at room temperature before the blots were detected in Electro-Chemi-Luminescence (ECL) solution (Pierce, USA) and Kodak film (Carestream Health, Rochester, USA). The developed films were scanned for data analysis.

### 2.6. Immunohistochemistry

Animals were anesthetized with 1.5% to 2% isoflurane and perfused transcardially. Lumbar spinal cord samples (L4–L6) and the injured sciatic nerve were removed. Lumbar spinal cord samples were postfixed for 4 h, then immersed in 30% sucrose dissolved in 0.01 M phosphate buffer. Transverse spinal sections (20 µm) were cut in a cryostat. Sections were blocked and then incubated with primary antibodies [mouse anti-GFAP, Cy3 conjugate, 1:400 (Millipore, USA)] overnight at 4 °C. The injured sciatic nerves were fixed in 4% paraformaldehyde for 3 to 6 h. Dehydrated, paraffin-embedded sections of 4 µm were soaked in xylene and rehydrated before immunostaining. Rehydrated samples were incubated at 95–100 °C for 10 min with sodium citrate buffer and then blocked, incubated with primary antibodies [rat antimyelin basic protein, 1:200 (Abcam, USA); rabbit anti-MMP9, 1:200 (Abcam, USA); mouse anti-β100, 1:300 (Abcam, USA); rat anti-Ly6g, 1:100 (Abcam, USA); goat anti-IL-1β, 1:50 (R&D Systems, USA)]. All antibodies were diluted in phosphate-buffered saline containing 5% fetal bovine serum for 12–16 h at 4 °C. The corresponding secondary antibodies [goat antirat conjugated to Alexa Fluor 488, 1:1000 (Invitrogen, USA); donkey antirabbit conjugated to Cy3, 1:500 (Millipore, USA); goat antimouse conjugated to FITC, 1:200 (Millipore, USA); donkey antigoat conjugated to Alexa Fluor 488, 1:1000 (Abcam, USA)] were incubated for 1 h at 4 °C in the dark. Then the sections were covered with mounting medium containing DAPI (Vector Laboratories, USA). Colocalization of MMP9 with β100, Ly6g, or IL-1β was evaluated by immunofluorescent staining. The images were captured with a Leica fluorescence microscope. GFAP- and Ly6g-positive cells were counted using Image-Pro Plus 6.0.

### 2.7. Statistical analysis

Data are presented as means ± SEMs and were analyzed using SPSS 16.0 or GraphPad Prism. The 50% withdrawal thresholds were compared among the 4 groups or across time points in one group using repeated-measures ANOVA or one-way ANOVA, followed by a post hoc LSD or Dunnett test, respectively. Differences between two groups were compared using unpaired t-tests. In all cases, differences of P < 0.05 were considered statistically significant.

## 3. Results

### 3.1. S1PR2 and pain-related gene expressions in a wild-type mouse model of neuropathic pain (Figures 1A–1H)

**Figure 1:**
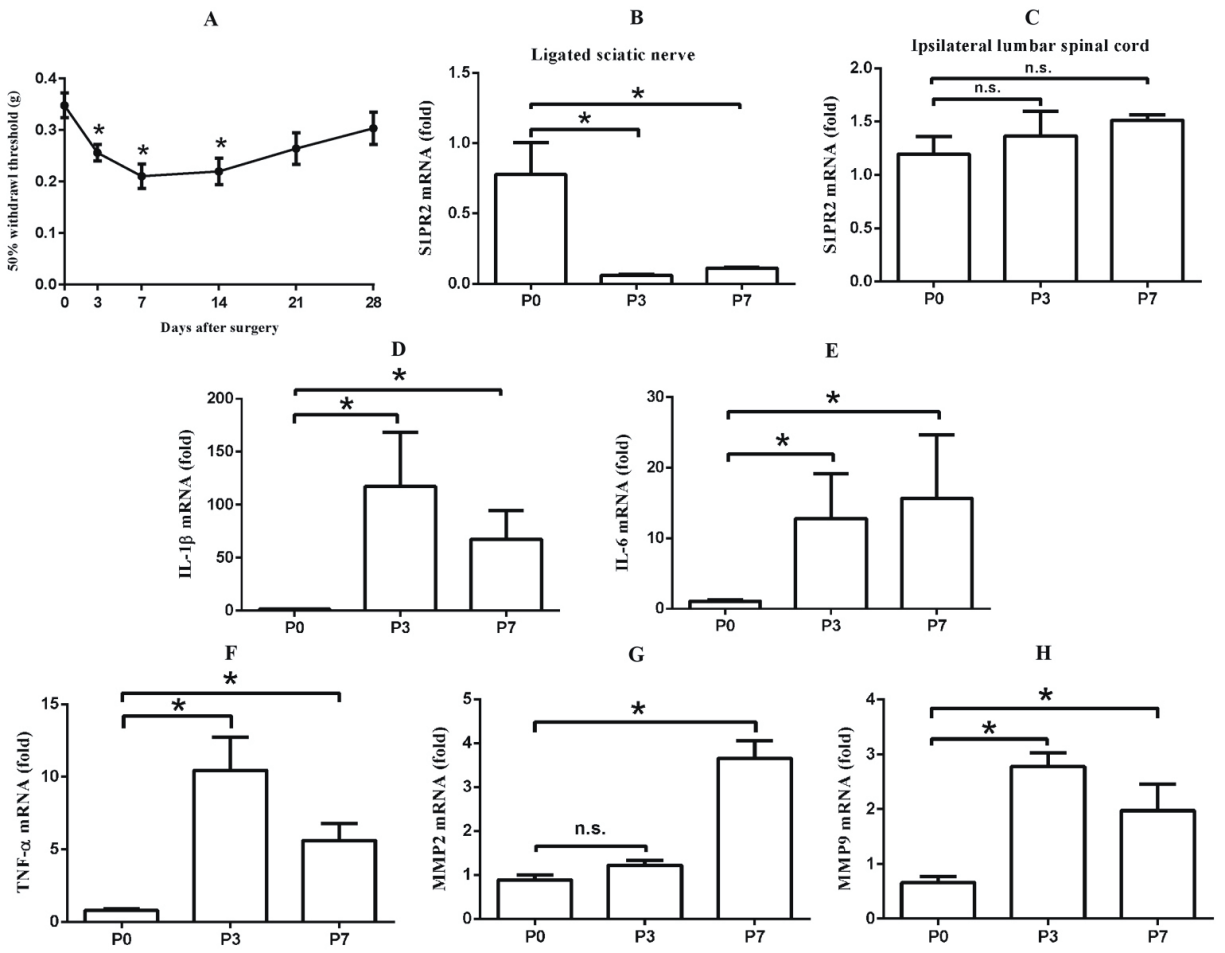
S1PR2 and pain-related genes expression in pSNL-induced NP mice. A) In WT NP mice, 50% withdrawal thresholds to mechanical stimuli were assessed (n = 6). *P < 0.05 vs. baseline value. B, C) S1PR2 expression in the wild-type NP model mice. S1PR2 mRNA expression was tested by RT-PCR in both the ligated sciatic nerve (B) and the ipsilateral lumbar spinal cord (L4–L6) (C) (n =5–6). *P < 0.05 vs. baseline value. D–H) Pain-related gene expressions of IL-1β, IL-6, TNF-α, MMP2, and MMP9 in the ligated sciatic nerve, respectively (n = 5–6). *P < 0.05 vs. baseline value. n.s., Not significant.

After partial ligation of the sciatic nerve, the 50% withdrawal thresholds on day 3, day 7, and day 14 after surgery showed significant differences from the baseline (P < 0.05) (Figure 1A). Pain-related gene expressions such as IL-1β, IL-6, TNF-α, MMP2, and MMP9 were increased after surgery (Figures 1D–1H). The reduced pain threshold and elevated pain-related gene expressions meant that the NP model had been generated successfully. Then S1PR2 expression was measured in the ipsilateral lumbar spinal cord and the injured sciatic nerve. In L4–L6, there was no difference in S1PR2 mRNA expression after surgery compared with the baseline (Figure 1C). However, in the ligated sciatic nerve, the S1PR2 mRNA expression after surgery was markedly decreased compared with the baseline (P < 0.05) (Figure 1B). These results showed that S1PR2 mRNA might be involved in the development of NP.

### 3.2. Neutrophil infiltration in injured nerves and astrocyte activation in lumbar spinal cord in a wild-type mouse model of neuropathic pain

**Figure 2:**
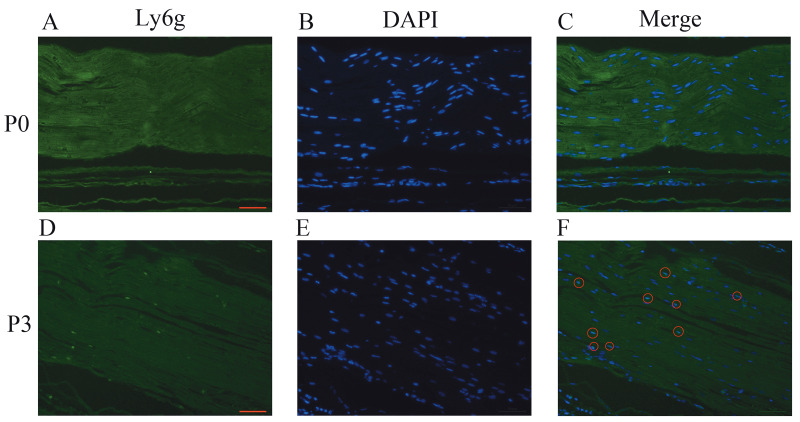
Neutrophil infiltration in the sciatic nerve in a wild-type mouse model. A, D) Ly6g-positive cells. B, E) DAPI, which marked the nucleic acids. C, F) Double immunofluorescence merged from the two pictures on the left. Red circles represent neutrophils. P0 means baseline. P3 means 3 days after pSNL. Scale bars: 50 μm.

**Figure 3:**
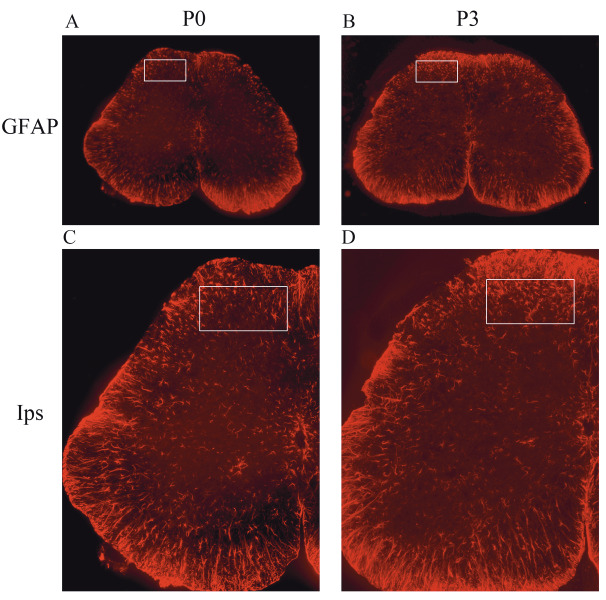
Astrocyte activation in lumbar spinal cord in a wild-type mouse model. Sections in the ipsilateral lumbar spinal cord were immunostained with an activated astrocyte marker (GFAP antibody, red). White boxes represent the ipsilateral dorsal horn. A and B are magnified by a factor of 40×. C and D are magnified by a factor of 100×. P0 means baseline. P3 means 3 days after pSNL. Ips means ipsilateral.

After partial ligation of the sciatic nerve in a wild-type mouse, Ly6g-positive cells (neutrophils) were observed near the injury site of the sciatic nerve at day 3 after pSNL (Figure 2). At the same time, the astrocytes (GFAP-positive cells) began to be activated in the ipsilateral dorsal horn (Figure 3). Consistent with previously reported studies (12,13), these results reconfirmed that the NP model had been generated successfully.

### 3.3. S1PR2-deficient neuropathic pain model mice show a decreased mechanical threshold

**Figure 4:**
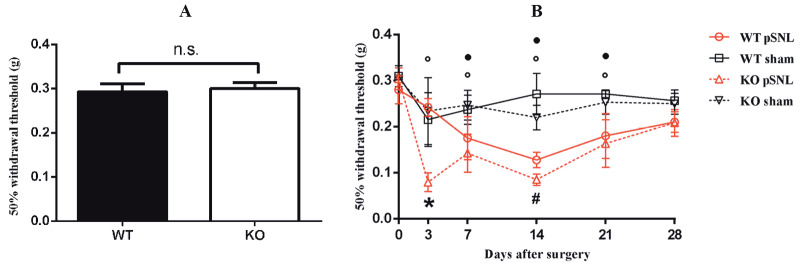
S1PR2 deficiency leading to a change in mechanical sensitivity. A) The physiological pain thresholds of WT and S1PR2-KO mice (n = 12). n.s., Not significant. B) The changes in 50% withdrawal threshold after surgery. *P < 0.05 between the S1PR2-KO surgery group and each of the other 3 groups. #P < 0.05 between each of the 2 surgery groups and their corresponding sham group. ○ P < 0.05 between the nerve-ligated group of S1PR2-KO mice and its own baseline value. ● P < 0.05 between the nerve-ligated group of WT mice and its own baseline value. Data are expressed as means ± SEMs. n = 5–6.

To assess whether S1PR2 deficiency alters physiological pain, we compared the nocifensive responses of WT and S1PR2-KO mice (produced by the heterozygous offspring intercrossing) to mechanical stimuli. In the von Frey filament test, there was no difference in the 50% withdrawal threshold of the hind paw upon mechanical stimulation between WT and S1PR2-KO mice (Figure 4A). Since there was no difference in pain under normal physiological conditions, we observed the mechanical threshold change after surgery. Figure 4B shows that after pSNL, the mechanical thresholds of the two surgery groups declined obviously (P < 0.05), while the mechanical thresholds of the two sham groups did not alter obviously compared with the baseline. When comparing each group at each time point, we found that at day 3 after surgery, the mechanical threshold of the S1PR2-KO group was significantly lower than those of the other 3 groups (P < 0.05). These results implied that S1PR2 could affect NP induced by pSNL.

### 3.4. Lack of influence of S1PR2 on cytokine and matrix metalloproteinase production in the damaged nerves

**Figure 5:**
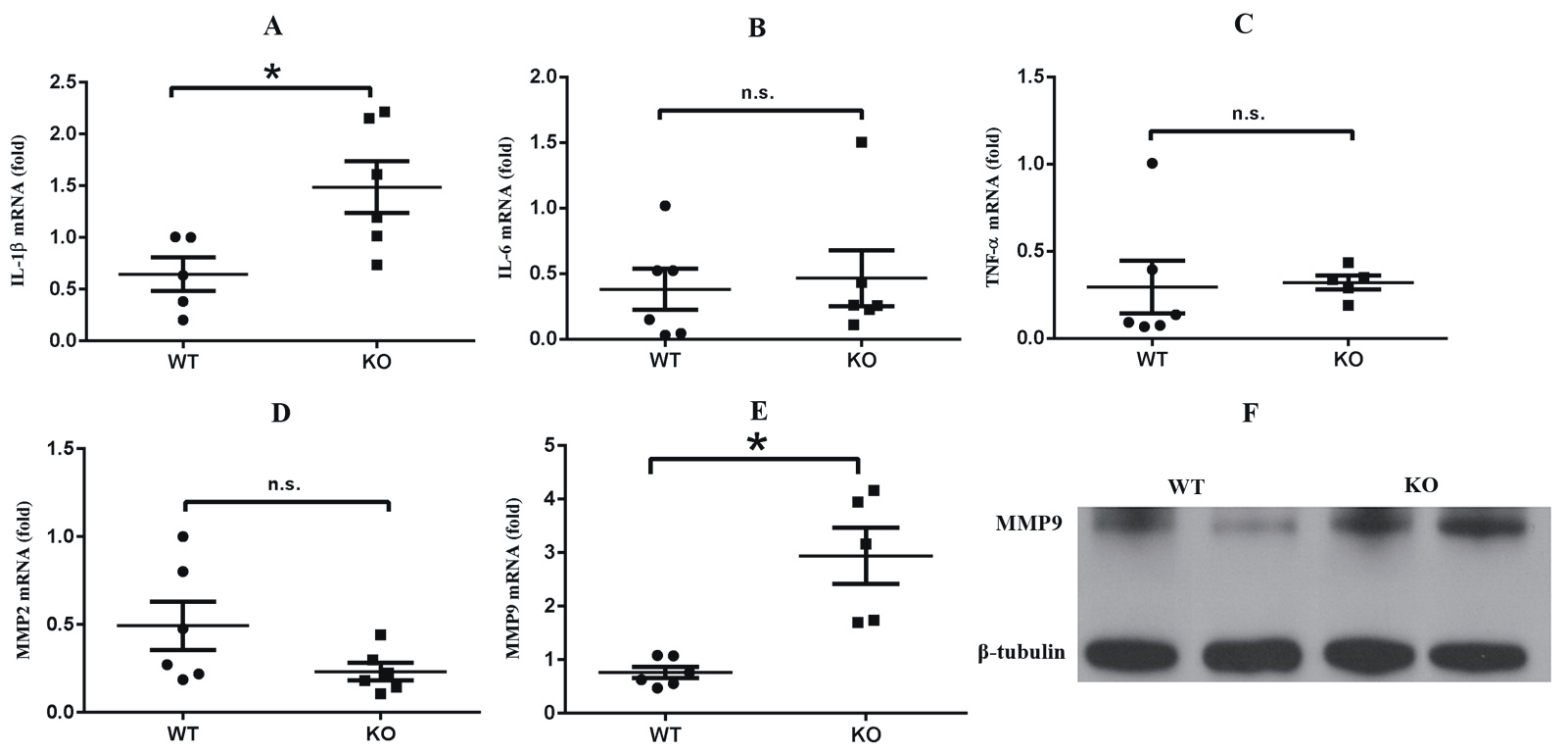
The expression of cytokines and matrix metalloproteinases in the damaged sciatic nerves at 3 days after surgery. A–E) mRNA expression of IL-1β, IL-6, TNF-α, MMP2, and MMP9, respectively. F) Representative western blot comparing the protein expression of MMP9 between 2 groups. *P < 0.05 between the WT group and the S1PR2-KO group. n.s., Not significant. Data are expressed as means ± SEM. n = 5–6.

Since depletion of S1PR2 resulted in the mechanical threshold decreasing at day 3 after surgery, we mainly focused on this time point to measure related proinflammatory cytokines and matrix metalloproteinases that are known, based on previous reports, to be present in increased amounts after pSNL (8,12–14). Thus, the mRNA levels of TNF-α, IL-6, IL-1β, MMP9, and MMP2 in the different groups of mice were examined using RT-PCR (Figures 5A–5E). Our results showed that IL-1β expression was significantly higher in S1PR2-KO mice than in WT mice. The other 2 cytokines presented no difference. MMP9 expression in S1PR2-KO mice was also significantly higher than that in WT mice. In addition, the results of a western blot for MMP9 yielded similar results (Figure 5F). These data implied that the change in mechanical sensitivity might be mediated by the elevation of IL-1β and MMP9 expression.

### 3.5. S1PR2 deficiency and neutrophil infiltration in injured nerves and astrocyte activation in lumbar spinal cord after partial sciatic nerve ligation

**Figure 6:**
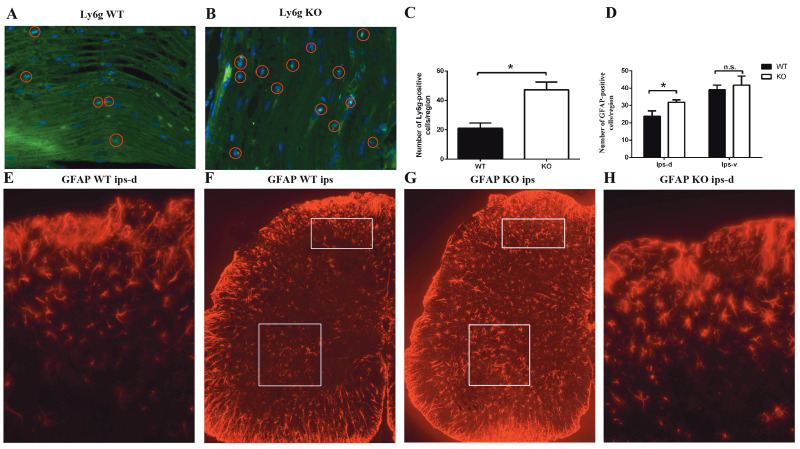
Neutrophil infiltration into injured sciatic nerves and astrocyte activation in the lumbar spinal cord after pSNL. A, B) Sections around the pSNL ligation site of WT and S1PR2-KO mice were immunostained with a neutrophil marker (Ly6g antibody, green) and a nucleic acid dye (DAPI, blue). Red circles represent neutrophils. The images are magnified by a factor of 400×. C) The number of Ly6g-positive cells in the sections were counted. D) The GFAP-positive cells in the sections were counted. E, H) The ipsilateral dorsal horn is shown, magnified by a factor of 400×. F, G) Sections in the ipsilateral lumbar spinal cord were immunostained with an activated astrocyte marker (GFAP antibody, red). White boxes represent the ipsilateral dorsal and ventral horn, where the cells were counted. The images are magnified by a factor of 200×. *P < 0.05. n.s., Not significant. Data are expressed as means ± SEM. n = 5.

To assess whether S1PR2 modulates inflammatory responses in a NP model, we carried out immunohistochemistry staining for different immune cells both in the peripheral ligated nerve and in the central lumbar spinal cord (L4–L6). An accumulation of Ly6g-positive cells was observed near the ligation site of the sciatic nerve at day 3 after pSNL. The number of positive cells was greater in S1PR2-KO mice than in WT mice (Figures 6A–6C). The number of activated astrocytes was greater in the ipsilateral dorsal horn of S1PR2-KO mice than in that of WT mice. However, there was no difference in the ipsilateral ventral horn. Furthermore, we found that astrocytes from S1PR2-KO mice developed thicker cell bodies and larger processes (Figures 6D–6H). This revealed that S1PR2 depletion could lead to more neutrophil infiltration and astrocyte activation.

### 3.6. Location of matrix metalloproteinase 9 in the peripheral sciatic nerve after surgery

**Figure 7:**
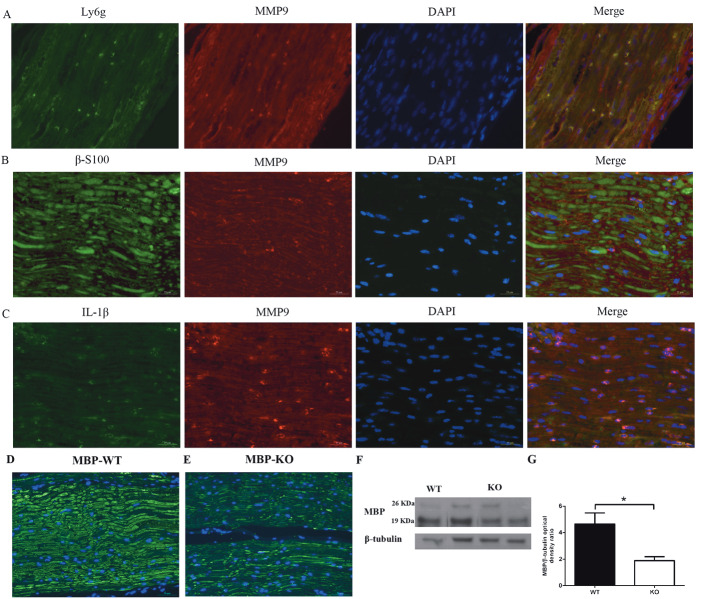
Localization of matrix metalloproteinase 9 in the peripheral sciatic nerve and expression of its substrate MBP. A–C) Green represents Ly6g, β-S100, and IL-1β. Red marks MMP9 and blue marks DAPI. The right-most picture shows triple immunofluorescence merged from the three pictures on the left. Scale bars: 25 μm. D, E) Sections of the injured sciatic nerves of WT and S1PR2-KO mice 3 days after pSNL were double immunofluorescence stained for MBP (green) and nucleic acids (DAPI, blue). The images are magnified by a factor of 400×. F, G) Representative comparison of MBP expression between the two groups by western blot. *P < 0.05. Data are expressed as means ± SEM. n = 4.

Schwann cells can produce MMP9, which modulates neuroinflammation, in response to nerve crush injury (14). Neutrophils can participate in the tissue’s inflammatory reaction by releasing MMP9 in the ischemic brain after occlusion/reperfusion (15). Since the MMP9 expression of S1PR2-KO mice was increased significantly at day 3 after surgery, we examined the localization of MMP9 by immunofluorescence. We found that the neutrophil marker Ly6g colocalized with MMP9 (Figure 7A), and the colocalization between MMP9 and the Schwann cell marker β-S100 was not obvious (Figure 7B). A previous report suggested that MMP9 could cleave IL-1β precursors to produce the mature form (16). In this study, we found that MMP9 and IL-1β were clearly colocalized (Figure 7C). These results implied that S1PR2 deficiency could lead to more neutrophil infiltration and MMP9 release, after which MMP9 would cleave IL-1β precursors into mature IL-1β.

### 3.7. Lack of influence of S1PR2 on myelin basic protein expression in the peripheral sciatic nerve

MMP9 plays a critical role in demyelination in the central and peripheral nervous systems (14,17). It can degrade myelin basic protein (MBP) synthesized by Schwann cells, which is critical for maintaining the integrity of the peripheral nerves (18–20). Therefore, we detected MBP expression in the injured sciatic nerve at day 3 after surgery. The MBP abundance in S1PR2-KO mice was significantly lower than that in WT mice. In addition, the immunofluorescence images showed consistent results (Figures 7D–7G). These data suggested that S1PR2 deficiency could cause an increase in MMP9 expression followed by more degradation of MBP.

## 4. Discussion

In this study, we first observed the relationship between the dynamic change in S1PR2 mRNA and the change in the pain threshold of WT mice with NP. As expected, S1PR2 mRNA expression in the injured nerve decreased after pSNL. Next, S1PR2-KO mice were used to study the role of S1PR2 in the formation of NP. The result showed that S1PR2 deficiency could reduce the mechanical threshold at day 3 after surgery compared with WT mice. Then we focused on this time point to seek the underlying cellular and molecular mechanisms. For S1PR2-KO mice, the number of activated astrocytes whose bodies became thicker and processes grew larger was increased in the central lumbar spinal cord; the number of infiltrated neutrophils was also increased in the peripheral ligated sciatic nerve. Next, proinflammatory cytokines and matrix metalloproteinases were measured. IL-1β and MMP9 expression levels were higher in the S1PR2-KO group than in the WT group. Since it has been reported that MMP9 can cleave the IL-1β precursor into the mature form (16), we observed the localization of MMP9 and its colocalization with IL-1β. Finally, the expression of MMP9’s substrate MBP was checked.

IL-1β is a highly proinflammatory cytokine that is produced as a result of inflammasome assembly, which activates the cysteine protease caspase-1. Caspase-1, in turn, cleaves the IL-1β precursor into the active form (21–24). Unpublished data from our group demonstrated that S1PR2 in macrophages under inflammatory stimulation causes inflammasome assembly and the production of active IL-1β. When S1PR2 is knocked out, the expression of active IL-1β declines. However, in this study, the depletion of S1PR2 led to elevated expression of IL-1β, which implied that the increase of IL-1β may not be due to the S1PR2-KO macrophages.

In addition to being cleaved by caspase-1, the precursor of IL-1β can also be cleaved by MMP9 and MMP2 (16). MMP9 and MMP2 belong to the family of matrix metalloproteinases, which are zinc ion- and calcium-dependent enzymes and have the ability to degrade the extracellular matrix (25). Increased expression of MMP9 and MMP2 was induced by peripheral nerve injury, which can destroy the basal lamina around the capillary, leading to the collapse of the vascular nerve barrier and the subsequent recruitment of more neutrophils from the blood (26). In the present study, the finding that MMP9 is elevated in S1PR2-KO mice is not in conflict with the increased IL-1β expression. MBP is the major structural protein of myelin, which mediates intracellular signal transduction through its interaction with actin and tubulin (27).

Because S1PR2 is widely expressed in the pain conduction pathway, we were unable to characterize the full spatial expression pattern of S1PR2, which will need to be confirmed in further experiments. Furthermore, the mechanisms of the increase in MMP9 expression after S1PR2 knockdown and its interaction with IL-1β are not well understood and will need further study in the future.

In conclusion, our results first indicate that S1PR2 deficiency in mice with NP could result in a decreased mechanical pain threshold through accumulated neutrophil infiltration and activated astrocytes, as well as elevated IL-1β/MMP9 production at the site of the nerve injury. S1PR2 may be a promising target for the treatment of NP.

## Acknowledgments

The authors extend their sincere thanks to Dr Yong-Xing Yao for animal model constructing, and to Professor Xiang-Yao Li for his technical support.
